# Laboratory information management software for engineered mini-protein therapeutic workflow

**DOI:** 10.1186/s12859-019-2935-x

**Published:** 2019-06-17

**Authors:** Mi-Youn Brusniak, Hector Ramos, Bernard Lee, James M. Olson

**Affiliations:** 1 Fred Hutchinson Cancer Research Center, 1100 Fairview Ave N, Seattle, WA 98109 USA; 2 LabKey Software, 617 Eastlake Ave E #400, Seattle, WA 98109 USA

**Keywords:** Laboratory information management system, Therapeutic protein, HPLC/UPLC peak classification, Protein engineering, LabKey software™

## Abstract

**Background:**

Protein based therapeutics are one of the fastest growing classes of novel medical interventions in areas such as cancer, infectious disease, and inflammation. Protein engineering plays an important role in the optimization of desired therapeutic properties such as reducing immunogenicity, increasing stability for storage, increasing target specificity, etc. One category of protein therapeutics is nature-inspired bioengineered cystine-dense peptides (CDPs) for various biological targets. These engineered proteins are often further modified by synthetic chemistry. For example, candidate mini-proteins can be conjugated into active small molecule drugs. We refer to modified mini-proteins as “Optides” (Optimized peptides). To efficiently serve the multidisciplinary lab scientists with varied therapeutic portfolio research goals in a non-commercial setting, a cost effective extendable laboratory information management system (LIMS) is/was needed.

**Results:**

We have developed a LIMS named Optide-Hunter for a generalized engineered protein compounds workflow that tracks entities and assays from creation to preclinical experiments. The implementation and custom modules are built using LabKey server, which is an Open Source platform for scientific data integration and analysis. Optide-Hunter contains a compound registry, in-silico assays, high throughput production, large-scale production, in vivo assays and data extraction from a specimen-tracking database. It is used to store, extract, and view data for various therapeutics projects. Optide-Hunter also includes external processing stand-alone software (HPLCPeakClassifierApp) for automated chromatogram classification. The HPLCPeakClassifierApp is used for pre-processing of HPLC data prior to loading to Optide-Hunter. The custom implementation is done using data transformation modules in R, SQL, javascript, and java and is Open Source to assist new users in customizing it for their unique workflows. Instructions for exploring a deployed version of Optide-Hunter can be found at https://www.labkey.com/case%20study/optide-hunter

**Conclusion:**

The Optide-Hunter LIMS system is designed and built to track the process of engineering, producing and prioritizing protein therapeutic candidates. It can be easily adapted and extended for use in small or large research laboratories where multidisciplinary scientists are collaborating to engineer compounds for potential therapeutic or protein science applications. Open Source exploration of Optide-Hunter can help any bioinformatics scientist adapt, extend, and deploy an equivalent system tailored to each laboratory’s workflow.

## Background

Following significant advancements in biologics and biopharmaceuticals, protein-based therapeutics has surpassed 10% of the entire pharmaceutical market and is expected to be an even larger proportion of the market in the future [1]. Peptide and protein drugs target a wide variety of therapeutic areas such as cancer, inflammation, endocrine, infectious diseases and more [2]. In the development of peptide and protein therapeutics, protein engineering is an essential part of achieving the desired therapeutic properties in terms of target specificity, stability, pharmacokinetics, pharmacodynamics, etc. Protein engineering is not limited to amino acid sequence alteration. Conjugation with small molecule (dye or drug) can be used to produce antibody drug conjugates (ADCs) or peptide-drug conjugates (PDCs) [3].

Tracking conjugations and other modification steps while manufacturing and producing therapeutic proteins is challenging because it involves many more processing steps than the small molecule high throughput design equivalent. These steps can be complex and it is crucial that the process steps are captured for repeatability, whether the engineered protein production is being performed in a GMP or GLP research lab, preclinical lab, or in an academic lab. It is also important to keep track of protein generation lineage for retrieval of data with related sequences, especially in high throughput engineering processes. Frequently, it is beneficial to search previously engineered proteins that possess sequence similarity. As an example, our laboratory investigates nature-inspired cystine-dense peptides (CDPs) that originated from spider, snake, grasshopper, and other species. We have made more than a thousand CDPs and characterized them based on their express-ability in our mammalian cell expression system [4]. We usually start from natural amino acid sequences (homologues) that are then modified to improve binding, serum half-life, and many other pharmacodynamic or pharmacokinetic properties (e.g., ^14^C-labelled peptides for autoradiography-based biodistribution or alanine scanning for structure activity relationship). During the sequence engineering, it is desirable to maintain the evolutionary lineage of the candidate CDPs from the natural homologue sequences so as to better inform further mutation or modification strategies.

There are several commercially available Laboratory Information Management Systems (LIMS) that can be used to address lineage and process tracking. Many can be configured as needed and some can be customized through software development. Our lab faced simultaneously building of a LIMS system while creating an experiment pipeline, as is common among academic research groups. Therefore, it was difficult to prepare reasonable software requirement specifications to establish ready-made/turn-key solutions up front. We found that the Open Source LabKey platform provided a budget-friendly and easily extendable and adaptable LIMS solution. LabKey is a well-documented Open Source platform for scientific data integration and analysis in a broad array of experimental settings [5]. This manuscript describes the customization of a LabKey server for application to our engineered peptide therapeutic candidates’ workflow. The customization includes our custom code for multiple Open Source modules with an Apache 2.0 license. The hope is that Optide-Hunter can assist other academic labs or small biotechnology organizations to jumpstart their protein engineering-based therapeutics workflows and easily adapt the provided code and example server for their unique needs. The modules introduced in this publication are free of charge to setup except for integration FreezerPro® connection. LabKey provides purchasable add-on special instrument connection packages and annual support if the users desire guidance from LabKey personnel rather than its user community.

## Implementation

### Protein engineering compound lineage tracking and customizable assay views for candidate therapeutics prioritization

**Fig. 1:**
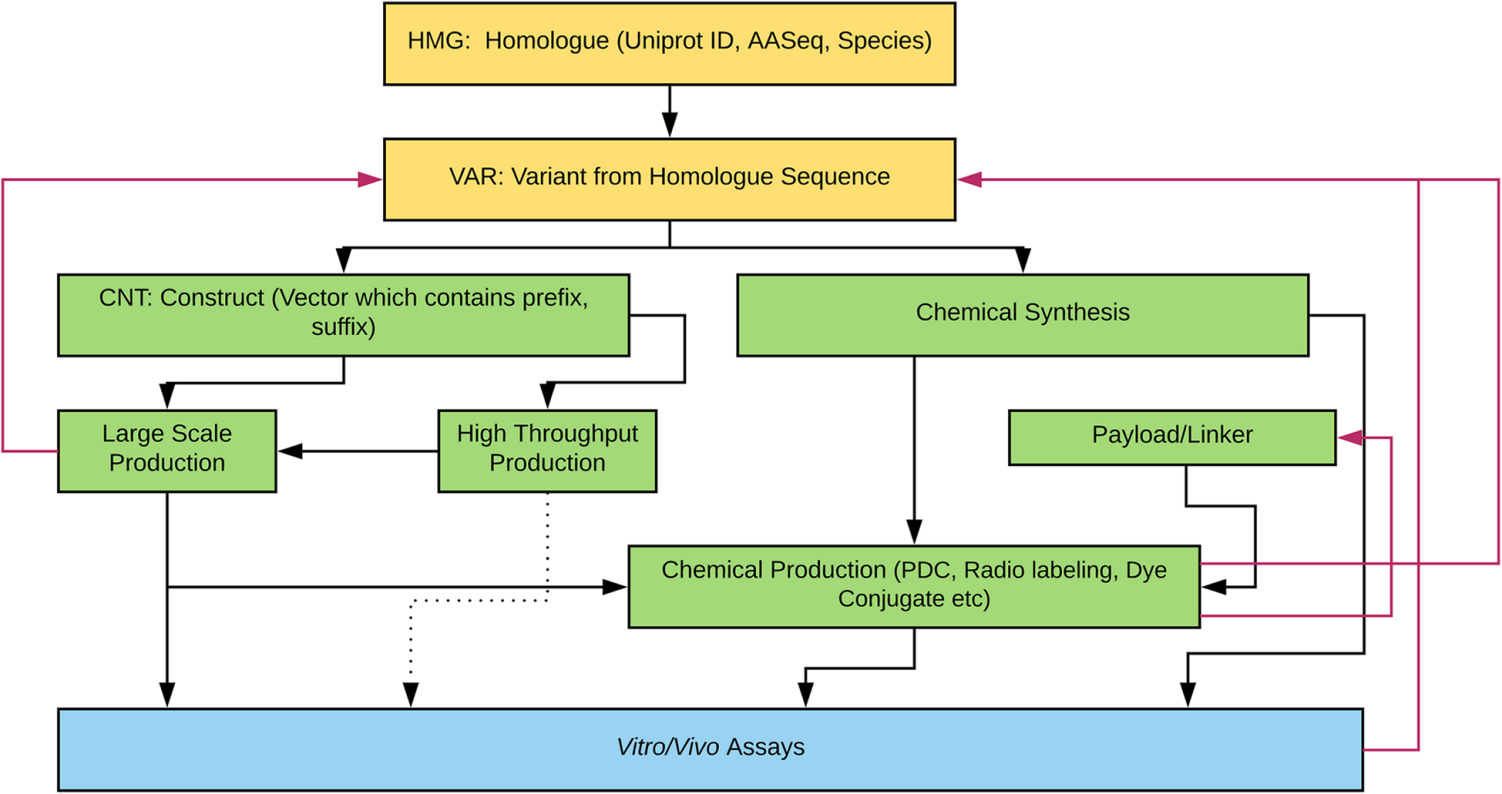
Protein Engineering Workflow. The bioinformatics data/literature mining with or without therapeutic targets is the starting point of root protein sequences. The software allows *in-silico* designed protein sequences as starting points as well as those with Uniprot designations. The majority of proteins we have explored are from sequences harvested from publicly available genomes. Thus, they have species and Uniprot numbers in the Homologue sample set database fields. Black arrows show the typical engineering paths. The dotted line from high throughput production is rare due to the amount of protein produced at this scale and current lack of efficient purification protocols. Red arrows indicate going back up the hierarchy to redesign proteins based on failures or other criteria (purity, express-ability by recombinant protein expression system, synthesizability etc.)

Figure 1 illustrates the various engineering pathways for therapeutic proteins. Compound registration starts with bioinformatics research and data mining for candidate peptides. Some of our compounds have a Uniprot number because they are native proteins produced by plants, animals, microbes, or other organisms. However, other compounds are de novo protein designs generated through the use of computational modeling software. From the parent sequences, variant sequences are registered. The variant sequence proteins can be chemically synthesized or be expressed by recombinant expression vector systems using bioengineering techniques. When the bioengineering platform is used to generate proteins, the construct sequences with prefix and suffix are added (e.g., enzyme cleavage site, polyhistidine-tag, etc). The construct can be used in either large scale (up to 10 mg/L in 2 L cell culture) or high-throughput scale (up to 20 μg in 1 mL scale 96 well plate culture). The proteins are screened by in vitro, ex vivo, or in vivo assays without further molecular structure modification. However, sometimes, the proteins are chemically modified (e.g., PDC) prior to biologic assays. Several properties (e.g., purity, express-ability by recombinant protein expression systems, synthesizability, etc.) are considered prior to progressing further along the drug discovery pipeline. Thus, based on predefined criteria, some sequences return to previous steps for redesign which are denoted by red arrows in Fig. 1.

**Fig. 2:**
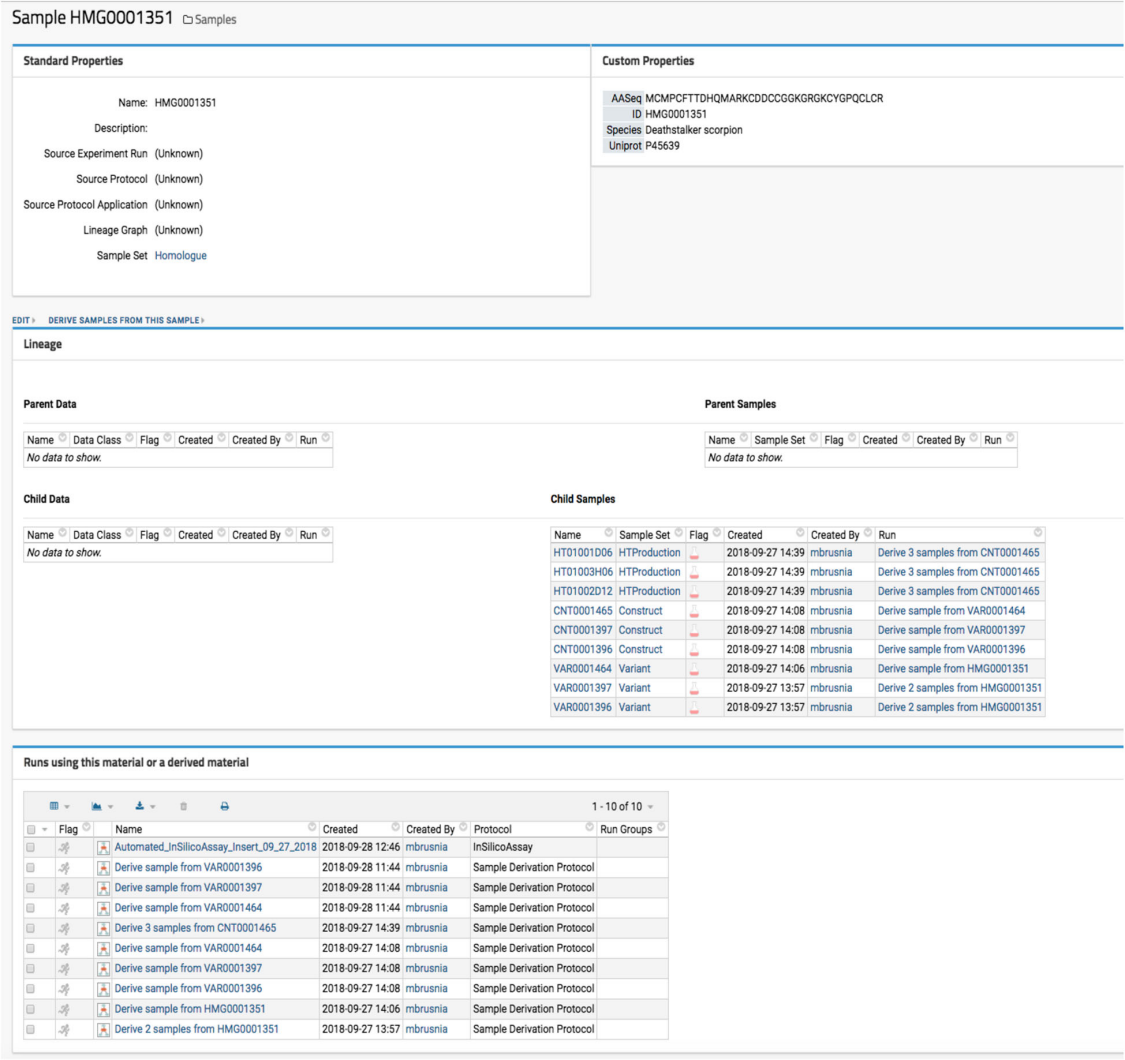
Retrieving Lineage. Both parent and child samples of a given engineered protein can be easily retrieved and displayed. This figure illustrates that the HMG0001351 has its three additional variance sequence children (VAR0001396, VAR0001397 and VAR0001464). Derivation is listed in the “Runs using this material or a derived material” table. All the blue words are hyperlinks for getting additional information

Optide-Hunter utilizes the LabKey “Sample Set” data container and “Parent Column” lookup field as a database foreign key constraint. This ensures that all sequences must have a valid parent ID to be accepted for registration. Thus, all sequences that are derived from registered parents can be reviewed as illustrated in Fig. 2.

**Fig. 3:**
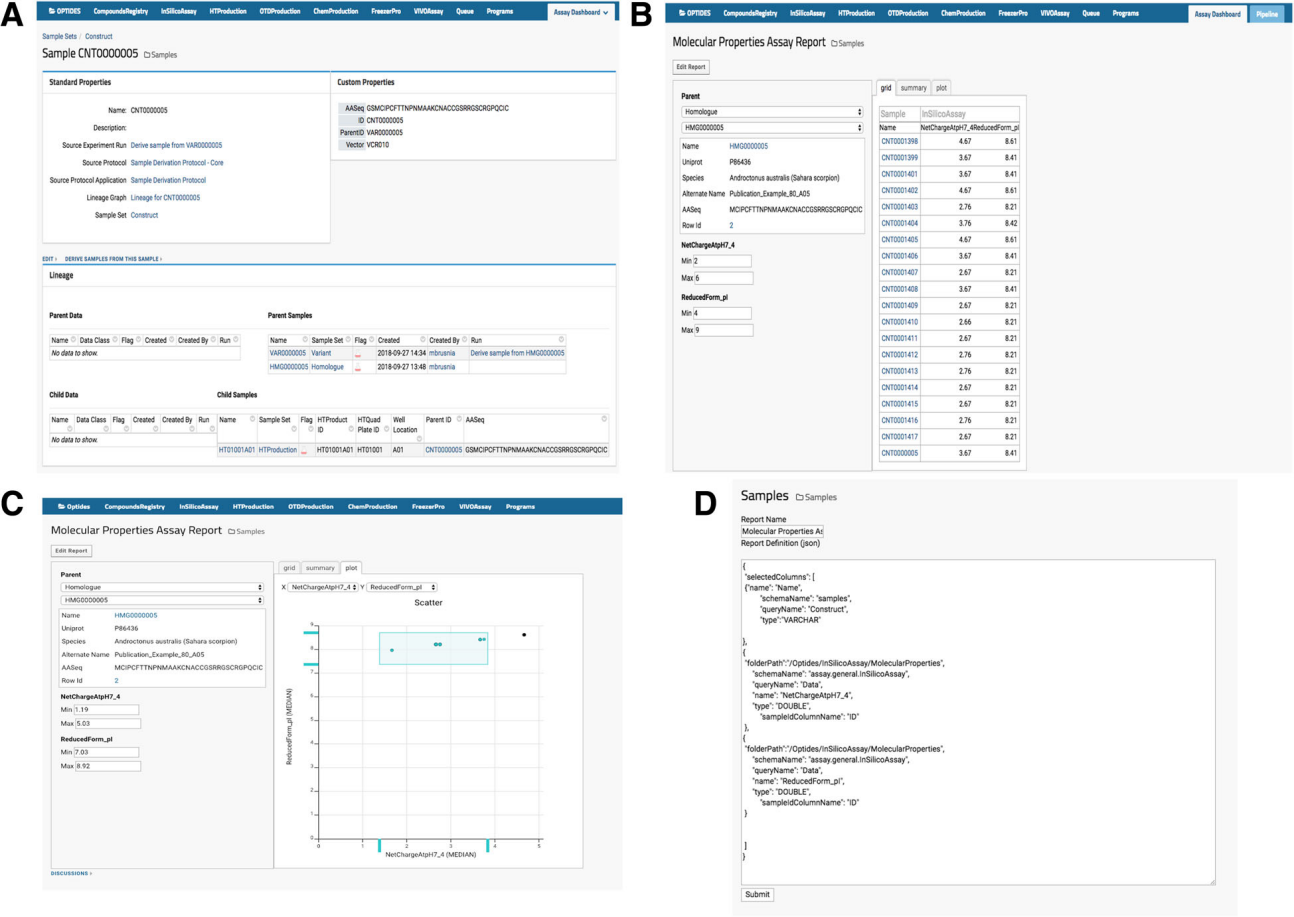
Engineered Protein Selection Module. (**a**) A new module called “AssayReport” is available as an optional module to the currently distributed modules in the compound registry page. (**b**) Upon parent compound selection, all variants from the parent are shown and also filtered by user specified property values or (**c**) filtered by selection of a specified region in the scatter plot for further selection of engineered proteins. (**d**) Administrators, developers or bioinformaticians can customize through the “Edit Report” function and adding or replacing JSON code as needed

Optide-Hunter also implemented a custom module called “AssayReport” and added it as an optional module to the list of other existing LabKey modules shown in Fig. 3A. This module provides the “Molecular Properties Assay Report” view that enables a user to filter child compound property values for comparison or prioritization in the therapeutics discovery pipeline as in Fig. 3B. The module also enables a user to filter through the graphical utility shown in Fig. 3C. The report can be easily customized through the “Edit Report” function in Fig. 3D by an administrator or system developer. The source code can be updated to add or replace sample and assay data as shown in Fig. 3D.

### Generalized assay collection pipeline and input transformation codes

**Fig. 4:**
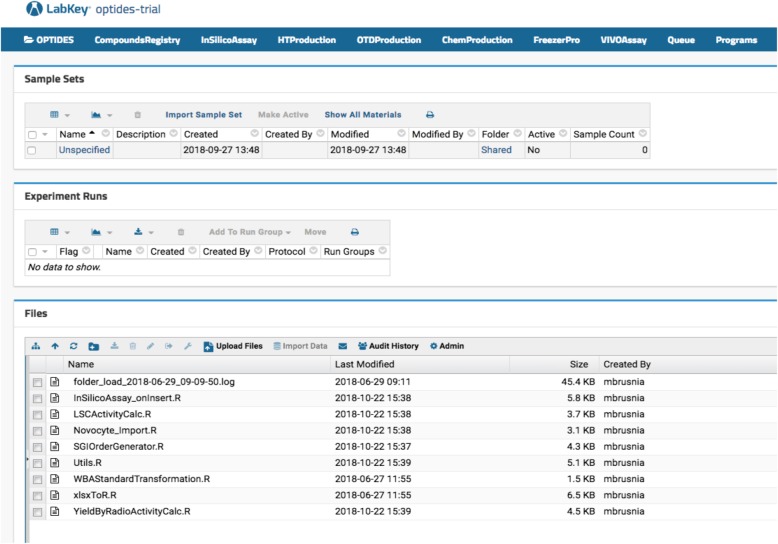
Data Transformation R codes. All code mentioned in the paper are embedded in the deployed LabKey customization interfaces such as folder schema. Our custom transformation R code resides in the top-level Optide project folder and is downloadable for further customization

Optide-Hunter server deployment has an in-silico function that calculates molecular properties of registered proteins. As shown in Fig. 1, our therapeutics discovery pipeline is not linear. However, proteins are generally designed and interrogated using in-silico assays, then manufactured by high throughput protein expression systems. Based on the high throughput assay data, large scale production is initiated and the peptide is then further modified chemically. Those intermediate and final products are stored in a third-party specimen tracking LIMS called FreezerPro®. Some of the compounds are further studied in vivo. In Optide-Hunter, the assay workflows can be identified using navigation menus configured in the LabKey header referred to as “InSilicoAssay”, “HTProduction”, “OTDProduction”, “ChemProduction”, “FreezerPro®” and “VIVOAssay” shown in Fig. 4. To capture the assay data in a useful way, we used the LabKey data transform functions on data input from Microsoft Excel files. This was done with R code installed in Optide-Hunter and shown in the “Files” panel in Fig. 4. For example, InSilicoAssay_onInsert.R parses tabular Excel files that are uploaded by a user and checks for duplicated sequences in order to avoid duplicate DNA synthesis orders. Next, molecular properties such as average mass, monoisotopic mass, net charge at pH 7.4, and hydrophobicity are calculated and inserted into the database. An R developer can customize the calculations by adapting the InSilicoAssay_onInsert.R source code. We customized the mass calculation for our CDP compounds because the disulfide bond formations of every cysteine site are important. The mass calculation accounts for the pair of hydrogen bonds lost at cysteine sites when disulfide bonds are formed. Similarly, WBAStandardTransformation.R parses Whole Body Autoradiography (WBA) data with radioactive concentration standards and uses the linear fit values to transform the raw data. The R transform script then uses the linear fit to calculate decay per minute (dpm) of a compound’s radioactivity in various tissues (brain, Xenograft tumor, blood, etc). These two examples are among several scripts that can be easily adapted for use with any R package or other major scripting language. The RLabKey package is essential for data retrieval and insertion into the underlying LabKey database.

### External pipeline for enhanced flexibility and faster data transformation

**Fig. 5:**
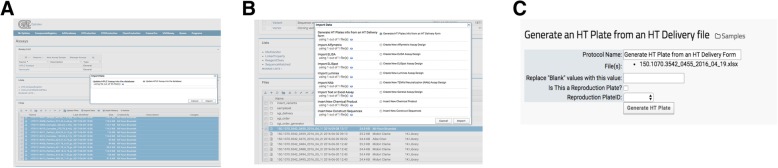
External Module Interface. (**a**) This example shows the use of multiple selected external HPLC assay files as input into the database. (**b**) This example shows HT Plate information retrieved from an HT delivery form external module selection. (**c**) Shows input parameters to run the HT plate generation module

When data processing requires additional functions, heavy computational demand, or user input parameters, invoking a LabKey external pipeline module provided flexibility that a transformation script (described in the previous section) cannot. The external pipelines, as opposed to the built-in processing pipelines, were built and deployed via Apache Tomcat, which is a necessary component of LabKey Server. The external module then runs as desired, reporting its status to the user: complete, running, or in error. Optide-Hunter currently deploys four such customized external pipelines. One loads 96 well plate HPLC data to our HTProduction Assay. A user selects the desired chromatography files and executes the corresponding “Import Data” option, “Update HPLC Assay into database”, shown in Fig. 5A. We have provided these custom modules which use standard LabKey module structure. The tasks and pipelines can be edited in the folder to easily create a new external pipeline. The insert_jpegs.R component file contains an algorithm to parse the chromatogram jpeg file names to find matched compounds in the database. It then populates corresponding property values in the HTProduction Assay database table. Optide-Hunter also uses an external pipeline module called “Generate HT Plates from an HT Delivery form” that creates HT barcodes automatically with delivered DNA information and inserts them into the database (Fig. 5B). In this case, the module accepts several user input parameters prior to processing (Fig. 5C).

### Project portfolio management and relevant data reports

**Fig. 6:**
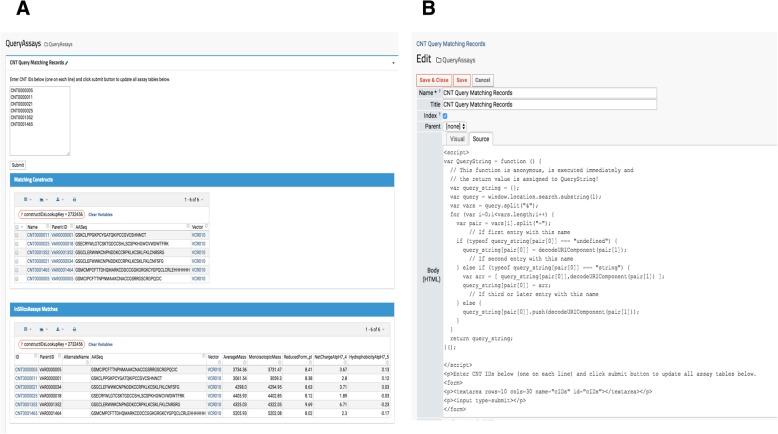
Custom Report to Retrieve Data Associated with Investigating Compounds. (**a**) Compound IDs retrieve all assay data associated with each ID along with parent compound information. (**b**) Administrators or developers can click the edit button (pencil icon) in the top box to see the source code schema

When a research lab manages several project portfolios for different therapeutic targets, querying for specific sample(s) information is important. While LIMS typically provide data querying services, interrogating similar or the same compound across multiple therapeutic target evaluations is quite difficult. Thus, a custom reporting module that gathers all relevant data from various assays (HTProduction, OTDProduction, CHEMProduction etc) becomes an indispensable tool for investigators. For example, a lab scientist generates data for one assay, but needs to simultaneously view another type of data. Similarly, project managers may only want to investigate promising subsets of samples. In our platform, a user submits a set of “Construct” sample IDs (Construct IDs) as keys to query the entire set of assays contained in our system. The resulting page’s URL contains the queried IDs so users can bookmark the entire URL containing the target therapeutic program compounds as shown in Fig. 6A. When new assay or production data associated with the queried construct IDs become available, it automatically populates. This feature is found under the “Programs” menu in the top banner and the source code is visible to administrators and developers by clicking “Edit Source” shown at the bottom in Fig. 6B.

### External software LIMS integration

**Fig. 7:**
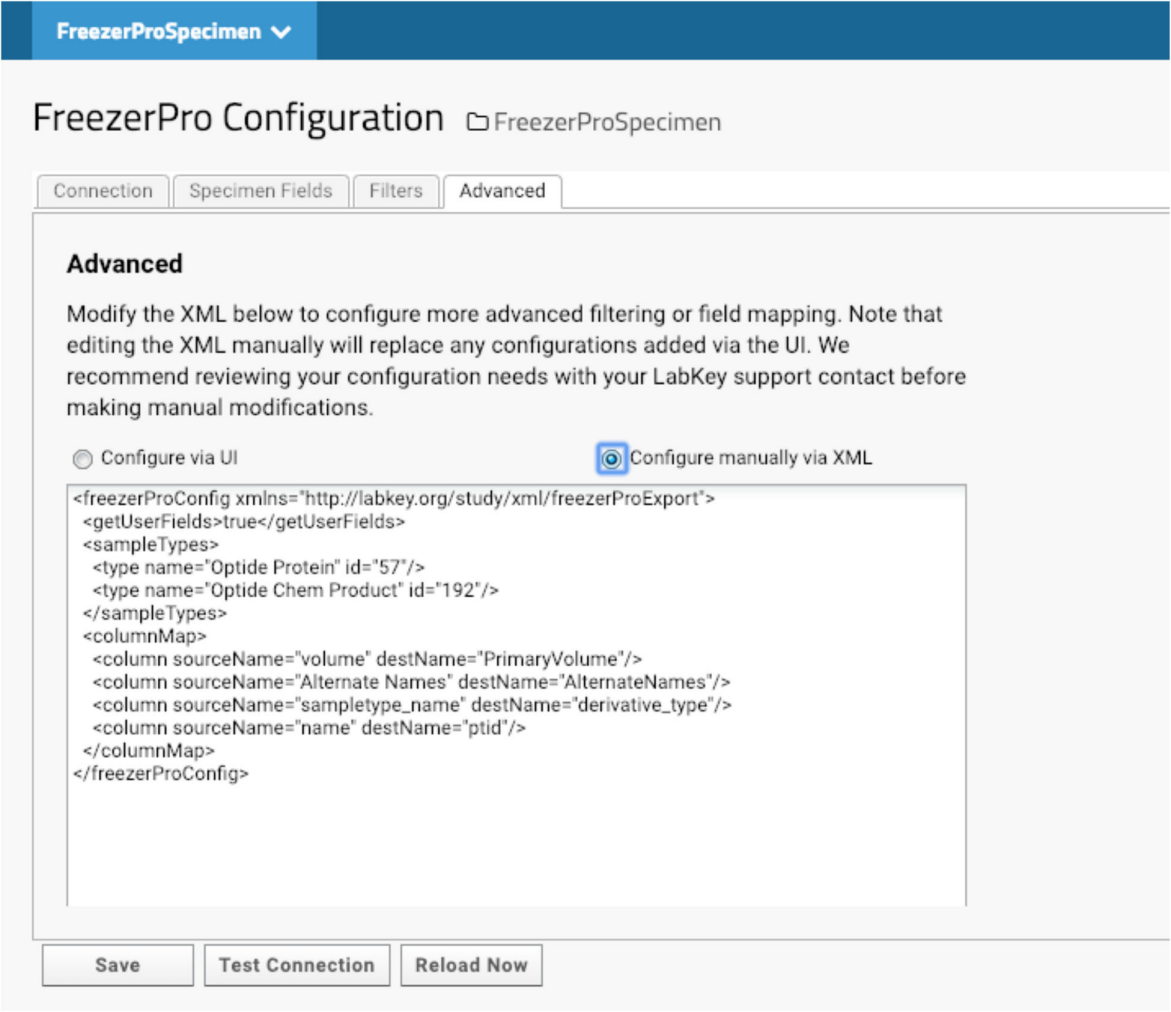
External LIMS Interface and Custom code. XML is used to configure filtering or mapping between Optide-Hunter and third party databases such as FreezerPro®

The compounds that we have made are deposited in -20F or -80F freezers. Specimen tracking is done through FreezerPro® which is commercially licensed on a per-user basis. Therefore, few lab scientists are authorized to access the specimens for accessioning or releasing. When compounds are produced, they are aliquoted to several vials for future use. Most of our lab users and program managers need limited information about each specimen to devise experiments, such as the total amount of each compound. They do not need to know non-research related information like the location of each specimen. We have implemented LabKey’s integration with FreezerPro® to provide filtered FreezerPro® data for users. (Fig. 7).

### Additional automation with scheduled tasks in windows operating system

Some assay tables contain data that need to be processed after other steps are taken and new related data become available. For example, one scientist may analyze a protein using gel electrophoresis and report their findings in the assay table in the LIMS. Then another lab scientist may run the same protein in liquid chromatography mass spectrometry (LC-MS) and report their findings in the same assay table. After the relevant data are populated in the database, the scheduled tasks are invoked to perform calculations and insert the results into a designated column in the assay table. For example, mass spectrometry m/z data is reported by a lab scientist. The scheduled task code is run overnight on the associated compound(s) and assigns a “true or false” validation status for the compound. More specifically, we calculate monoisotopic mass with full disulfide bond formation and compare it with various charge states. If the measured m/z is one of the available values, the scheduled script ascribes a status of “true” to the compound. The frequency (minutes, hours, or days) of the scheduled job is easily adjusted through Microsoft Window Operating System task scheduler. The scripts utilize the RLabKey package in R and can be easily customized for the needs of another lab.

### Liquid chromatography peak classifier module

**Fig. 8:**
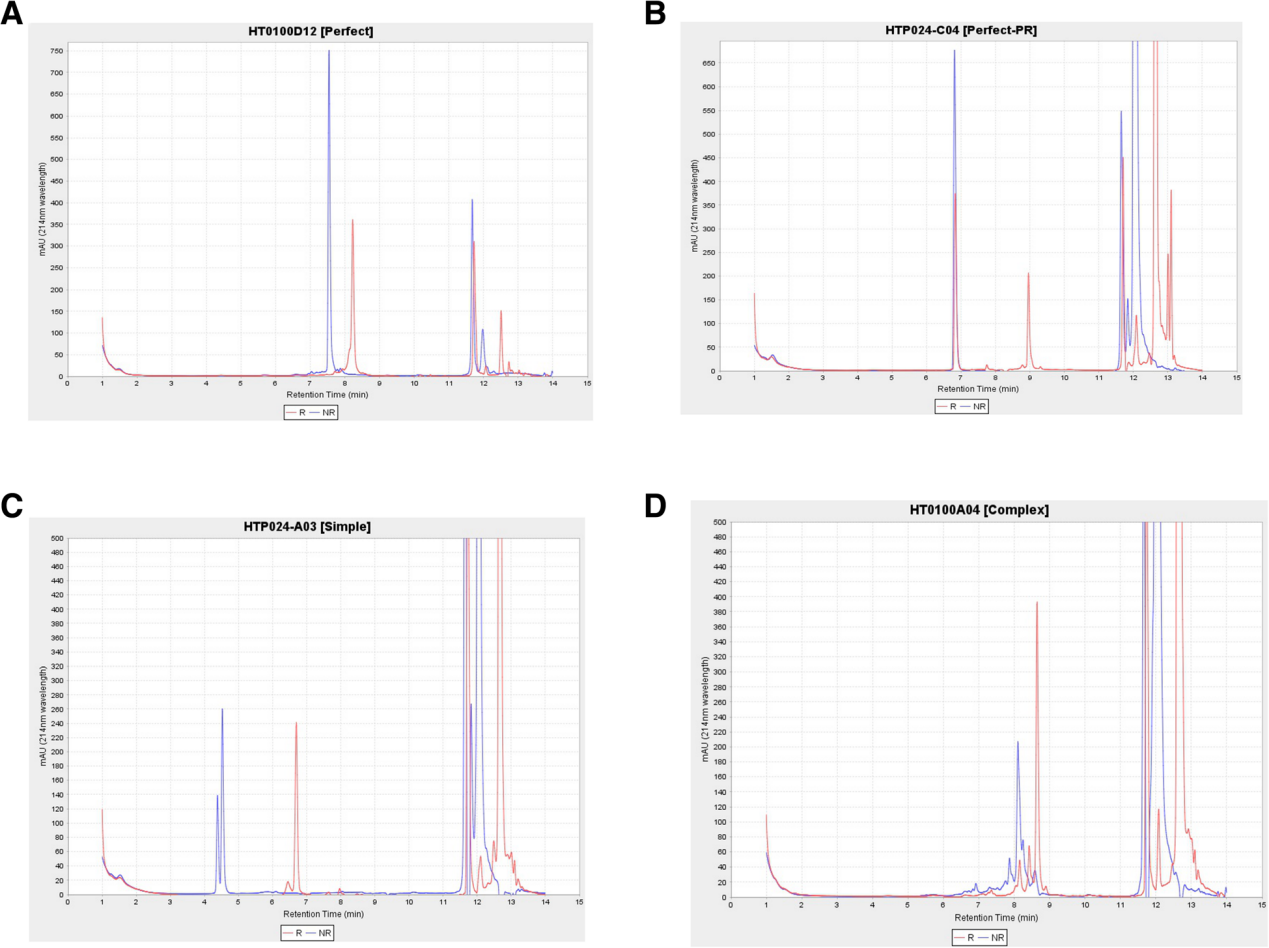
HPLCPeakClassifierApp Classification. The blue trace is from intact protein without DTT treatment and the red trace is from the same protein with DTT treatment. The number of peaks is identified after blank sample normalization provided that the trace is greater than user defined signal-to-noise ratio. (**a**) Each protein is classified as “Perfect” when there is one blue trace and one shifted red trace that indicates high protein purity with disulfide bond formation. (**b**) Protein is classified as “Perfect-Partial” when there is a single peak in the blue trace and the red trace has two peaks of which one overlaps with the blue trace, indicating that disulfide bond formation is partially reduced. This type of protein can be of particular therapeutic interest since it shows higher resistance to DTT reduction, which implies that the peptide may remain intact in the typical reductive intracellular environment. (**c**) Protein is classified as “Simple” when either the blue or red trace has two peaks including shoulder peaks. (D) Protein is classified as “Complex” when either the blue or red trace has more than two peaks

This workflow also contains stand-alone modules by a custom application intended to automatically provide quality scores for liquid chromatography (LC) based measurements. Bioengineered expressed proteins are run through either Agilent high performance liquid chromatography (HPLC) or by Waters Ultra performance liquid chromatography (UPLC) for trace characteristics. In addition to a general trace assessment of protein production, CDPs require an additional disulfide bond formation assessment. More specifically, CDPs are a promising molecular class due to their particular arrangement of disulfide bonds in their core that provide structural stability. This signature disulfide bond formation can be a critical attribute, for example, in the development of an orally delivered therapeutic compound. Thus, for each produced protein, two LC traces are obtained: one trace is from intact purified protein and the other trace is the Diethothreitol (DTT) treated protein. With an overlaying of the two traces, the protein is classified as (1) perfect (2) perfect-partial reduction (3) simple and (4) complex (Fig. 8). In order to classify compounds’ biophysical properties in a few defined categories in a consistent manner, we developed a method and stand-alone software (HPLCPeakClassifierApp). Our method involves a blank sample being run every three sets of pairs of protein samples (DTT treated and DTT not treated pair). All sample UV absorption trace values are normalized by subtracting the low noise values of the preceding blank sample.

The HPLCPeakClassifierApp has various input parameters for each lab to classify the traces that align with their research objectives. For example, the signal to noise ratio (−SN) value controls how many peaks are found within user defined retention time (RT) ranges (−MinRTForPeak, −MaxRTForPeak). By providing RT range as an input parameter, labs can change the values to accommodate different solvent gradients. The classification referred to as “Simple” is a more subjective classification that heavily depends on the screen objectives and can be stringent or permissive. Thus, a parameter called “-Classification” is provided to define number of acceptable peaks to be classified as “simple”.

## Results

The compilation of integration and automation efforts has produced Optide-Hunter software, which is composed of pluggable R, SQL, HTML, JSON and Java code. It is a webserver-based LIMS and stand-alone LC processing toolset for engineered protein therapeutics discovery including in-silico design, bioengineered proteins, synthetic chemical modification, high throughput plate-based in vitro and animal model-based in vivo systems. Optide-Hunter is an actively deployed system that continuously collects data for repeatability and collaboration among our scientists who are in different physical locations. We provide here, access to a deployed web version of our system with fake sample data for the community to evaluate. We provide Open Source versions of our software for those who desire it, to adapt them for their workflow by modifying or extending the code and database table design. The instructions for evaluation can be found at https://www.labkey.com/case%20study/optide-hunter and all of the Optide code is embedded in the LabKey custom platform. We also packaged source codes in one place for easy retrieval by bioinformaticians or software engineers.

## Conclusion

Therapeutic development based on engineered protein platforms has been gaining ground in many disease indication fields. However, academic labs or start-up companies face two challenges in obtaining a useful LIMS. Often the workflow platform itself is under construction and it is hard to generate solid software requirement specifications up front. Furthermore, the cost of commercial LIMS can be prohibitive. This paper addresses the unmet need for those labs that require cost-effective and flexible LIMS for early stage experimental pipeline development for engineered protein therapeutics development.

## Data Availability

Project name: Optide-Hunter. Project home page: https://www.labkey.com/case%20study/optide-hunter Operating system(s): Clouds Hosting in Windows 10. Programming language: Java, JSON, R. Other requirements: NA. License: Apache License Version 2.0. Any restrictions to use by non-academics: No.

## Abbreviations

ADC: Antibody Drug Conjugates
HPLC: High-Performance Liquid Chromatrography
LC-MS: Liquid Chromatography-Mass Spectrometry
LIMS: Laboratory Information Management System
PDC: Peptide-Drug Conjugates
UPLC: Ultra Performance Liquid Chromatography
WBA: Whole Body Autoradiography
